# Brain Training Game Improves Executive Functions and Processing Speed in the Elderly: A Randomized Controlled Trial

**DOI:** 10.1371/journal.pone.0029676

**Published:** 2012-01-11

**Authors:** Rui Nouchi, Yasuyuki Taki, Hikaru Takeuchi, Hiroshi Hashizume, Yuko Akitsuki, Yayoi Shigemune, Atsushi Sekiguchi, Yuka Kotozaki, Takashi Tsukiura, Yukihito Yomogida, Ryuta Kawashima

**Affiliations:** 1 Smart Ageing International Research Centre, Institute of Development, Aging and Cancer, Tohoku University, Sendai, Japan; 2 Division of Developmental Cognitive Neuroscience, Institute of Development, Aging and Cancer, Tohoku University, Sendai, Japan; 3 Department of Functional Brain Imaging, Institute of Development, Aging and Cancer, Tohoku University, Sendai, Japan; 4 Japanese Society for the Promotion of Science, Tokyo, Japan; University of Groningen, The Netherlands

## Abstract

**Background:**

The beneficial effects of brain training games are expected to transfer to other cognitive functions, but these beneficial effects are poorly understood. Here we investigate the impact of the brain training game (Brain Age) on cognitive functions in the elderly.

**Methods and Results:**

Thirty-two elderly volunteers were recruited through an advertisement in the local newspaper and randomly assigned to either of two game groups (Brain Age, Tetris). This study was completed by 14 of the 16 members in the Brain Age group and 14 of the 16 members in the Tetris group. To maximize the benefit of the interventions, all participants were non-gamers who reported playing less than one hour of video games per week over the past 2 years. Participants in both the Brain Age and the Tetris groups played their game for about 15 minutes per day, at least 5 days per week, for 4 weeks. Each group played for a total of about 20 days. Measures of the cognitive functions were conducted before and after training. Measures of the cognitive functions fell into four categories (global cognitive status, executive functions, attention, and processing speed). Results showed that the effects of the brain training game were transferred to executive functions and to processing speed. However, the brain training game showed no transfer effect on any global cognitive status nor attention.

**Conclusions:**

Our results showed that playing Brain Age for 4 weeks could lead to improve cognitive functions (executive functions and processing speed) in the elderly. This result indicated that there is a possibility which the elderly could improve executive functions and processing speed in short term training. The results need replication in large samples. Long-term effects and relevance for every-day functioning remain uncertain as yet.

**Trial Registration:**

UMIN Clinical Trial Registry 000002825

## Introduction

Our cognitive functions change over our lifetimes [1], [2]. The elderly may experience a decline in a number of cognitive functions, including memory [2], [3], attention [4], executive functions [5], [6], processing speed [7]. Decline in cognitive abilities has been shown to lead to difficulty performing basic activities of daily living [1], [8]–[10]. Although our cognitive functions decline with age, some previous study showed that the brain retains some plasticity with age [11]–[13]. In fact, previous studies showed that several cognitive training programs could improve cognitive functions such as memory [14], [15], processing speed [16]–[18], executive function [19], and attention [20] in the healthy elderly. Thus, one of major goal of aging research is to develop methods for maintaining and improvement of cognitive functions for the elderly.

Video game training is one type of cognitive training in the elderly [21], [22]. Some previous studies showed that playing a video game could lead to improve some cognitive functions in the healthy elderly [23]–[26]. Because companies have been attracted to these results which playing video games could improve some cognitive functions in the health elderly, many types of brain training games (e.g. Brain Age, Big Brain Academy, and Brain Challenge) have been released. Since the brain training games were first released, they have been extremely popular around the world. The beneficial effects of these brain training games are expected to improve cognitive functions (e.g. executive function, memory, attention, processing speed), which is commonly referred to as a transfer effect. A transfer effect is that training has an effect not only on skills or performance that are trained, but also on skills or performance that are not trained. Based on the previous studies using video games [23], [27], [28], we simply defined the transfer effect as improvements on untrained cognitive performance from playing the brain training games. Yet in all honesty, the beneficial effects of the brain training games have little scientific basis [29], [30]. Previous studies have shown that the elderly persons have demonstrated that participating in cognitive training programs [14], [19] and playing certain types of games [23], [24] can improve cognitive functions. Although these results suggest that the brain training games could potentially improve the cognitive functions in elderly persons, scientific evidence for the beneficial effects of the brain training games on other cognitive functions in the elderly is still scarce.

Our goal for this study was to investigate the beneficial effect of a brain training game in the healthy elderly. To examine this issue, we adopted the brain training game called Brain Age published by Nintendo in 2005. Brain Age is one of the most popular brain training games. This game was developed based on the previous cognitive training for the elderly. Some previous studies [22], [23] suggested that video game training studies should include an active control group that plays other types of video game. Based on the suggestion, the active control group was designed to control for test-retest effects and positive effects to play some video games. Because previous studies used the Tetris as an active control group [31], we included the active control group that played the Tetris published by Nintendo in 2006.

To reveal the impact of the brain training game (Brain Age) on cognitive functions in the elderly, we conducted a double-blinded intervention. Participants and testers were kept blind to the experimental hypothesis. Participants in both the Brain Age and the Tetris groups played each game for about 15 minutes per day, at least 5 days per week, for 4 weeks.

To evaluate the effects of the brain training game, we assessed a broad range of cognitive functions. The measurements of the cognitive functions included four categories (global cognitive statuses, executive functions, attention and processing speed). Global cognitive statuses was measured by Mini-Mental State Examination (MMSE) [32]. Executive functions were measured by Frontal Assessment Battery at bedside (FAB) [33], and Trail Making Test-B (TMT-B) [34]. Attention was measured by Digit Cancellation Task (D-CAT) [35], Digit Span Forward (DS-F) [36], and Digit Span Backward (DS-B) [36]. Processing speed was measured by Digit Symbol Coding (Cd) [36] and Symbol Search (SS) [36].

## Materials and Methods

### Randomized controlled trial design

This study was registered in the UMIN Clinical Trial Registry (UMIN 000002825). This randomized controlled trial was conducted between March 2010 and August 2010 in Sendai city, Miyagi prefecture, Japan. Written informed consent to participate in the study was obtained from each participant. The protocol of this study and informed consent were approved by the Ethics Committee of the Tohoku University Graduate School of Medicine. The protocols for this study and supporting CONSORT checklist are available as supporting information (Checklist S1, Protocol S1 and Protocol S2).

To assess the impact of the brain training game on the elderly, we used a double blinded intervention. Participants and testers were blind to the study's hypothesis. Participants were blind to the treatment and control designations of these two groups, and were informed only that the study was designed to investigate the effects of two different training programs. Testers were blind to the group membership of participants. The researcher (N.R.) randomly assigned participants to either of two groups (Brain Age, Tetris) by the random draw using a computer.

### Participants

Forty-four participants were recruited through an advertisement in the local newspaper and screened by a questionnaire before inclusion. 12 participants declined to participate. All included participants (n = 32) reported to be right-handed, native Japanese speakers, not concerned about their own memory functions, not using medications known to interfere with cognitive functions (including benzodiazepines, antidepressants or other central nervous agents), and having no diseases known to affect the central nervous system, including thyroid disease, multiple sclerosis, Parkinson's disease, stroke, severe hypertension or diabetes. To maximize the benefit of the intervention, all participants were non-gamers and reported playing less than one hour of video games a week over the past 2 years [23], [37]. To minimize the influence of subclinical degenerative conditions, the following exclusion criteria were employed. Mini Mental Status Exam (MMSE)<26 [32], Frontal Assessment Battery at bedside (FAB)<12 [33] and IQ<85 derived from the Wechsler Adult Intelligence Scale III [36]. None of the participants were excluded on the basis of these.

**Figure 1:**
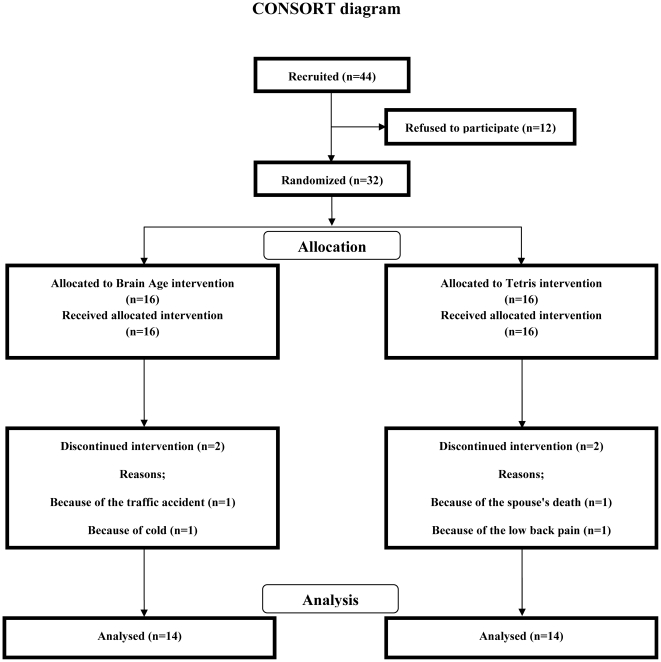
CONSORT flowchart.

All participants provided informed consent to participate in this study, which was approved by the Ethics Committee of the Tohoku University Graduate School of Medicine. After the informed consent was obtained, participants were randomly assigned to either of two groups (Brain Age, Tetris) by the random draw using a computer. The study was completed by 14 of the 16 members in the Brain Age group and 14 of the 16 members in the Tetris group (Figure 1). Table 1 summarizes the baseline demographics and neuropsychological characteristics of the participants included in the analyses. We found no significant differences between the groups on the demographics or neuropsychological characteristics. We did not use any types of a priori matching methods (e.g. nearest neighbor or frequency matching). After the random assignment, the sex, age and education level (years) in the Brain Age group were similar to the Tetris groups (Table 1). The scores of MMSE and FAB in the present study were similar to that in the previous community-dwelling the elderly studied in Japan [19], [38], [39].

**Table 1:** Characteristics of the participants in Brain Age and Tetris group.

	Brain Age group		Tetris group			
	(6M/8F)		(7M/7F)			
	Mean	SD	Mean	SD	Effect size (*d*)	*p*-value
Age (year)	68.86	(2.07)	69.31	(2.82)	.18	0.14
Education (year)	13.43	(2.38)	13.36	(2.13)	.03	0.67
IQ (score)	114.54	(14.72)	113.29	(13.66)	.09	0.66
MMSE (score)	28.50	(1.16)	28.50	(1.51)	.00	0.76
FAB (score)	14.04	(2.40)	14.00	(1.66)	.02	1.00

There are no significant difference between Brain age and Tetris groups (two sample t-test, *p*>0.10). M, the number of men; F, the number of women; MMSE, mini-mental state examination; FAB, frontal assessment battery at bedside; SD, standard deviation.

### Overview of intervention

The participants were asked to perform each video game training (Brain Age or Tetris) over 4 weeks with 5 training days in each week. On each training day, participants performed the video game for about 15 minutes. The participants played video games on the portable console, Nintendo DSi, at their homes. Game performance was recorded for each participant. At the end of each training day, participants reported the scores of the played games. The Brain Age group listed the titles of trained games and a score for each trained game at the end of each training day. The Tetris group only reported the best total score at the end of each training day. The measures of cognitive functions were conducted before and after training. On the first day of training (pre), all participants were tested on a series of neuropsychological and behavioral tests. After these tests, participants received the instruction to play one of the games for 30 minutes. To play the video game, participants were provided the portable console (Nintendo DSi) and one of the video games (Brain Age or Tetris). The following day, participants started 4 weeks video game training. After 4 weeks of training (post), all participants were re-examined on some neuropsychological and behavioral tests. Finally, the portable console and the video game were returned at the end of the study. The procedures for this study were approved by the Ethics Committee of the Tohoku University Graduate School of Medicine.

#### Brain training group (Brain Age)

We used Brain Age published by Nintendo as a game which participants in the brain training group played. Brain Age is one of the popular brain training games. It was developed based on the previous findings of a cognitive training program for the elderly [19]. The previous study used reading aloud and simple arithmetic calculations as training tasks. The reasons why these tasks were selected were that 1) these tasks activated the dorsolateral prefrontal cortex activated in comparison to the resting state [40], [41], 2) these task were very simple. Most games in Brain Age contain the elements of these reading aloud and simple arithmetic calculations.

Brain Age published by Nintendo has nine games. We used 8 training games with the exception of Voice Calculation, because Voice Calculation is similar to Calculation X 20 and Calculation X 100. 1) In Calculation X 20, participants are required to answer a total of 20 simple arithmetic calculations as quickly as possible. The questions include addition, subtraction, and multiplication. 2) In Calculation X 100, participants are required to answer a total of 100 questions as quickly as possible. The questions include addition, subtraction, and multiplication. 3) In Reading Aloud, participants are required to read excerpts from Japanese classical literature aloud. 4) In Syllable Count, some sentences written in a combination kanji and kana are presented. Participants are required to count the total number of kana letters after translating kanji to kana. 5) In Low to High, numbers in boxes are firstly presented for a few seconds. Then, participants are required to select the boxes from the lowest number to the highest number. 6) In Head Count, participants watch scenes in which some people enter or leave a house. Then participants are required to answer the number of people in the house at the end. 7) In Triangle Math, three numbers are presented on a top line (e.g. 5, 7, 2), two mathematical operations on a second line (e.g. +, +) and one mathematical operation (e.g. +) on a last line are presented. Firstly, participants are required to solve the first formula (5+7) using the first two numbers (5, 7) in the first line and the first mathematical operation (+) in the second line, and then the second formula (7+2) using the last two numbers (7, 2) in the first line and the last mathematical operation (+) in the second line. Then, participants are required to solve the last formula using the answer of the first formula (12), the answer of the last formula (9) and the mathematical operation (+) in the last line. In this case, participants give the final answer (21). 8) In Time Lapse, two analog clocks are presented. Participants are then required to calculate the difference in time between the two clocks. At the beginning of the game, participants can do only three trainings (Calculation X 20, Calculation X 100, and Reading Aloud). New games are added to the game list after training for some days. After playing the games, game performances of each game and the playing time of each game are recorded in the video game (Brain Age). We used these actual game performances to check that playing the game improved the performances of the trained games.

Participants received the following instructions. 1) Participants were asked to train for 15 minutes a day, five times a week during the 4 weeks. 2) Participants were required to play the Calculate X 20, the Calculate X 100 and the Reading aloud games on each training day. 3) When a new training game was available, participants could play the new game. 4) Participants were restricted from playing the Brain Age Check because this game mode included a task similar to the TMT [34], which is one measure of cognitive functions. 5) After each training day, participants were required to check the name of the played training games and to write down their high score of played training in a training diary. Although the actual game scores were recorded in the game, to keep the motivation to participate in this study, we asked participants to write down the game performances. After the intervention period, we checked whether or not the scores which participants reported matched the actual scores which were recorded in the video game. Although the most scores which participants reported were consistent with the actual scores, we used the actual game scores from the game in our analysis.

#### Active control group (Tetris)

We used Tetris published by Nintendo as a game which participants in the active control group played. Tetris is a popular puzzle game in which players rotate and move blocks descending from the top of the screen so that these blocks form lines at the bottom of the screen. After a complete line with no gaps is formed, the line disappears. If no lines are formed, the blocks pile higher and higher until the block pile reaches the top of the screen, at which point the game ends and the player loses. The goal is to keep the game going as long as possible by forming complete lines. As the game progresses, the blocks descend faster, giving players less time to choose where to place each block. After playing the game, game performance (total score) is recorded in the video game (Tetris). We used these actual game performances to check that playing the game improved the performances of the trained games.

Participants received the following instructions. 1) Participants were asked to train for 15 minutes a day, five times a week during 4 weeks. 2) After each training day, participants were required to write down the highest score they achieved while playing in a training diary. Although the actual game scores were recorded in the game, to keep the motivation to participate in this study, we asked participants to write down the game performances. After the intervention period, we checked whether or not the scores which participants reported matched the actual scores which were recorded in the video game. Although the most scores which participants reported were consistent with the actual scores, we used the actual game scores from the game in our analysis.

The Tetris group was designed to control for the positive effects that could be attributed to participating in this intervention study, such as using a computer and playing a game. Because 1) previous studies used the Tetris as an active control group [31] and 2) previous studies showed that playing the Tetris did not have transfer effects to other cognitive functions [24], we included the active control group that played the Tetris published by Nintendo in 2006.

### Overview of cognitive function measures

To evaluate the effects of the brain training game, we assessed a broad range of the cognitive functions. The measures of the cognitive functions fell into four categories (global cognitive status, executive function, attention, and processing speed). Global cognitive statuses was measured by Mini-Mental State Examination (MMSE) [32]. Executive functions were measured by Frontal Assessment Battery at bedside (FAB) [33], and Trail Making Test-B (TMT-B) [34]. Attention was measured by Digit Cancellation Task (D-CAT) [35], Digit Span Forward (DS-F) [36] and Digit Span Backward (DS-B) [36]. Processing speed was measured by Digit Symbol Coding (Cd) [36]and Symbol Search (SS) [36]. Details of all tasks were described below.

#### MMSE

MMSE [32] is the most widely used screening instrument for the detection of cognitive impairment in older adults. MMSE is a 20-item instrument. The items of MMSE measure orientation for place and time, memory and attention, language skills, and visuospatial abilities. MMSE is scored from 0 to 30. Lower scores of MMSE indicate greater degrees of general cognitive dysfunction. The primary measure is the total score of this task (max = 30).

#### FAB

FAB [33] evaluates executive functions. FAB consists of six subtests, namely, those for similarities (conceptualization), lexical fluency (mental flexibility), motor series (programming), conflicting instructions (sensitivity to interference), go–no go (inhibitory control), and prehension behavior (environmental autonomy). FAB is scored from 0 to 18. Lower scores of FAB indicate greater degrees of executive dysfunction. The primary measure is the total score of this task (max = 18).

#### TMT-B

TMT has been employed widely as a measure of executive function. TMT consists of two parts (TMT-A and TMT-B). TMT-A requires participants to link in ascending order series of 25 numbers (1–2–3 …) randomly distributed in space. TMT-B is similar, although instead of just linking numbers the subject must alternately switch between a set of numbers (1–13) and a set of letters (A–L), again linking in ascending order (1–A–2–B …). We used only TMT-B in this study. The primary measure of this test is the amount of time (seconds) required to complete the task. The score of TMT-B measures executive function.

#### D-CAT

D-CAT [35] evaluates attention. The test sheet consists of 12 rows of 50 digits. Each row contains 5 sets of numbers 0 to 9 arranged in random order. Thus any one digit would appear 5 times in each row with randomly determined neighbors. D-CAT consists of three such sheets. Participants were instructed to search for the target number(s) that had been specified to them and to delete each one with a slash mark as fast and as accurately as possible until the experimenter sent a stop signal. There were 3 trials, first with a single target number (6), second with two target numbers (9 and 4), and third with three (8, 3, and 7). Each trial was given 1 minute, hence the total time required for D-CAT was 3 minutes. In the second and third trials, it was stressed that all of the target numbers instructed should be cancelled without omission. The primary measure of this test is the number of hits (correct answers). We used only the number of hits in the first trial.

#### DS

DS is a subtest in WAIS-III [36]. This test evaluates attention. Digit Span has two subsections (DS-F and DS-B). For DS-F, participants repeat numbers in the same order as they were read aloud by the examiner. For DS-B, participants repeat numbers in the reverse order of that presented aloud by the examiner. In both, the examiner reads a series of number sequences in which the examinee is required to repeat the sequence in either forward or reverse order. DS-F has sixteen sequences. DS-B has fourteen sequences. The primary measures of this test are raw scores which refers to the number of correctly repeated sequences until the discontinue criterion (i.e., failure to reproduce two sequences of equal length) was met [36]. The maximum raw score of DS-F is 16. The maximum raw score of DS-B is 14.

#### Cd

Cd is a subtest of WAIS-III [36]. This test measures processing speed. For Cd, the participants are shown a series of symbols that are paired with numbers. Using a key, the participants draw each symbol under its corresponding number, within a 120 second time limit. The primary measure of this test is the number of correct answers.

#### SS

SS is a subtest of WAIS-III [36]. This test measures processing speed. The SS contains 60 items. For this subtest, the participants visually scan two groups of symbols (a target group and a search group) and indicate if either of the target symbols matches any of the symbols in the search group. The participants respond to as many items as possible within a 120 second time limit. The primary measure of this test is the number of correct answers.

### Data Analysis

**Figure 2:**
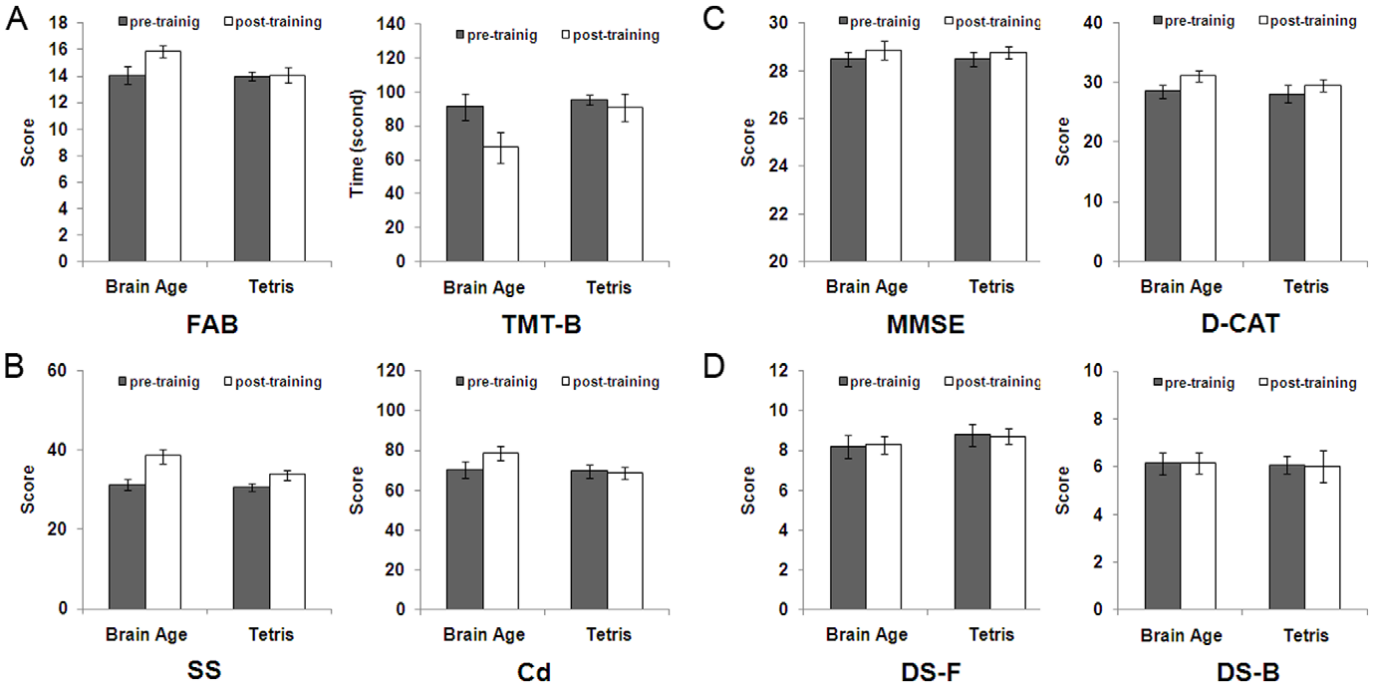
Cognitive function scores at before and after training in both groups. The group comparison (two sample *t*-tests) of the pre training scores demonstrated that there were no significant differences in any measures of cognitive functions between the brain training group and the Tetris training group (*p*>0.10). Error bars indicate SEM across subjects in each subject group. (A) The Executive functions were measured by frontal assessment battery at bedside (FAB) and trail making test type B (TMT-B). (B) The processing speeds were measured by symbol search (SS) and digit symbol coding (CD). (C) (D) The general cognitive function was measured by mini-mental state examination (MMSE). The attention was measured by digit cancellation task (D-CAT), digit span forward (DS-F) and digit span backward (DS-B).

The goal of this study was to evaluate the effect of the brain training game of the elderly. We calculated the change in score (post-training score minus pre-training score) in all measures of cognitive functions. We conducted multivariate analyses of covariance (MANCOVA) for the change scores (post-training score minus pre-training score) in each of cognitive tests (Figure 2, Table 3). The change scores were the dependent variable, groups (Brain Age, Tetris) was the independent variable. Pre-training scores in all cognitive tests, sex, age, and education levels (years) were the covariate to exclude the possibility that any pre-existing difference of measure between groups affected the result of each measure and adjust for background characteristics. The level of significance was set at *p*<0.05. Moreover, we report eta square (*η^2^*) as an index of effect size. It is a standardized difference in the change score (post-training score minus pre-training score) between intervention groups (Brain Age, Tetris). *η^2^*≥.01 is regarded as small effect, *η* ^2^≥.06 as medium effect, and *η^2^*≥.14 as large effect [42]. The group comparison (two sample *t*-tests) of the pre training scores demonstrated that there were no significant differences in any measures of cognitive functions between the brain training group and the Tetris training group (*p*>0.10, Figures 2).

## Results

As shown in Fugure 1, 32 participants participated in this study, and participants were randomized into the two groups (Brain Age and Tetris). The study was completed by 14 of the 16 members in the Brain Age group and 14 of the 16 members in the Tetris group. Before analyzing the transfer effects of the brain training game to other cognitive functions, we examined whether the practice improved the trained games. This analysis is the same as the previous studies which investigated the beneficial effects of the video game trainings [23], [37]. Participants in both groups showed significant improvement of the game performance in the last time playing than in the first time playing (paired *t*-test, *p*<0.05, Table 2).

**Table 2:** First and last game scores in both Brain Age and Tetris training groups.

		Pre	Post	Effect size (*d*)	*p*-value	Training days	Maximum training days
Tetris training group							
Total score (score)	M	1480.21	18862.50	1.21	0.00	19.78	20
	SD	(2697.68)	(20107.08)			(0.43)	
Brain Age training group							
Calculations X 20 (second)	M	54.57	25.86	1.82	0.00	19.43	20
	SD	(20.72)	(8.25)			(1.28)	
Calculations X 100 (second)	M	243.14	142.07	2.04	0.00	19.14	20
	SD	(55.43)	(42.98)			(1.75)	
Reading Aloud (word/minute)	M	5.71	9.93	1.51	0.00	19.57	20
	SD	(1.14)	(3.79)			(0.94)	
Low to High (score)	M	18.69	34.08	2.06	0.00	16.00	19
	SD	(9.02)	(5.50)			(4.91)	
Syllable Count (second)	M	205.23	113.38	1.55	0.00	14.64	18
	SD	(73.17)	(40.32)			(5.20)	
Head Count (score)	M	2.08	4.69	2.88	0.00	13.00	17
	SD	(1.19)	(0.48)			(5.92)	
Triangle Math (second)	M	131.23	70.38	1.27	0.00	5.14	7
	SD	(51.27)	(47.12)			(2.54)	
Time Lapse (second)	M	215.92	160.92	0.64	0.04	3.21	5
	SD	(89.66)	(81.50)			(2.22)	

There are significant differences between first and last game scores in all training of Brain Age and in Tetris (paired *t*-test, *p*<0.05). In the Brain Age group, the maximum number of training days on each training game was different because the training games were added to the training list through training. Pre, pre-training; post, post-training; M, mean; SD, standard deviation.

The goal of this study was to evaluate the effect of the brain training game of the elderly. To evaluate the transfer effect of the brain training game on the improvement of other cognitive functions, we conducted multivariate analyses of covariance (MANCOVA) for the change scores (post-training score minus pre-training score) in each of cognitive tests (Figure 2, Table 3). The change scores were the dependent variable. Group (Brain Age, Tetris) was the independent variable. Pre-training scores in all cognitive tests, sex, age, and education levels (years) were the covariate to exclude the possibility that any pre-existing difference of measure between groups affected the result of each measure and adjust for background characteristics.

**Table 3:** The score of change in cognitive functions measures of both groups.

	Brain Age Group		Tetris Group			
	Mean	SD	Mean	SD	Effect size (*η^2^*)	*p*-value
Executive function						
FAB (score)	1.79	(1.58)	0.07	(1.21)	0.13	0.001
TMT-B (seconds)	−24.00	(22.81)	−4.57	(22.32)	0.13	0.006
Attention						
D-CAT (number)	2.57	(4.36)	1.43	(3.11)	0.06	0.277
DS-F (low score)	0.07	(1.94)	−0.07	(1.86)	0.00	0.717
DS-B (low score)	0.00	(1.41)	−0.07	(1.90)	0.00	0.683
Global cognitive status						
MMSE (score)	0.36	(1.28)	0.29	(1.33)	0.00	0.631
Processing speed						
Cd (number)	8.29	(7.03)	−0.93	(8.08)	0.19	0.005
SS (number)	7.43	(4.91)	3.21	(5.13)	0.12	0.014

Change scores were calculated by subtracting the pre-cognitive measure score from the post-cognitive measure score. The Executive functions were measured by frontal assessment battery at bedside (FAB) and trail making test type B (TMT-B). The processing speeds were measured by digit symbol coding (Cd) and symbol search (SS). The global cognitive status was measured by mini-mental state examination (MMSE). The attention was measured by digit cancellation task (D-CAT), digit span forward (DS-F) and digit span backward (DS-B). We report eta square (η^2^) as an index of effect size. It is a standardized difference in the change score (post-training score minus pre-training score) between intervention groups (Brain Age, Tetris). *η^2^*≥.01 is regarded as small effect, *η* ^2^≥.06 as medium effect, and *η^2^*≥.14 as large effect. SD means standard deviation.

Results of these MANCOVAs for change scores showed that the effects of playing Brain Age were higher than that of playing Tetris in the all measures of the executive function (FAB, *F* (1, 12) = 17.16, *η^2^* = 0.13, *p* = 0.001); TMT-B, *F* (1, 12) = 11.16, *η^2^* = 0.13, *p* = 0.006) and to two measures of the processing speed measures (SS, *F* (1, 12) = 8.22, *η^2^* = 0.12, *p* = 0.014; Cd, *F* (1, 12) = 11.74, *η^2^* = 0.19, *p* = 0.005). These results indicated that effects of playing Brain Age were transferred to the executive function and the processing speed. However, there were no significant differences between effects of playing Brain Age and Tetris in a measure of the global cognitive statuses (MMSE, *F* (1, 12) = 0.24, *η^2^* = 0.00, *p* = 0.63) and all measures of the attention (D-CAT, *F* (1, 12) = 1.30, *η^2^* = 0.06, *p* = 0.28; DS-F, *F* (1, 12) = 0.14, *η^2^* = 0.00, *p* = 0.72; DS-B, *F* (1, 12) = 0.18, *η^2^* = 0.00, *p* = 0.68). These results suggested that playing the Brain Age did not improve the global cognitive statuses and the attention.

## Discussion

The present results are to demonstrate the transfer effect of Brain Age on the improvement of executive functions and processing speeds in the elderly. Given that both executive functions and processing speed decrease with age [7], [43] and that these functions are strongly correlated with the activities of daily life [44], one of main goals in the field of cognitive training for the elderly is to improve executive functions and processing speed. Our study has two important implications in the field of cognitive training for the elderly. Firstly, training periods (15 minutes per day, 4 weeks) in our study is shorter than that in previous cognitive training studies. Secondly, our study is to show improvements of executive functions and processing speed in the elderly. These results suggest that there is a possibility which the elderly could improve executive functions and processing speed in short term training. The results need replication in large samples. Long-term effects and relevance for every-day functioning remain uncertain as yet.

The findings are consistent with previous evidence that performing some cognitive training [19], [21] and playing certain games [23], [24] could contribute to the improvement of cognitive functions in the elderly. The mechanism of this transfer effect identified in our study could be explained by a recent hypothesis, which proposes that the transfer effect could be induced if the processes during both training and transfer tasks are overlapped and are involved in similar brain regions [45]–[47]. Most training games in Brain Age consist of processes necessary in the calculations and reading aloud. To perform these processes successfully, the prefrontal regions should be recruited [40], [41]. The executive functions and processing speed, which showed a significant transfer effect by the brain training game in our study, are also involved in the prefrontal cortex [48]. These findings suggest that both training games and transfer tasks could share the same brain region, prefrontal cortex, and that the transfer effect of the brain training game on the executive functions and processing speed could be mediated by the prefrontal regions. Moreover, another explanation of the transfer effect in our study is the combination hypothesis, in which the combination of components involved in training tasks could be important in the transfer effect [22], [49]. In our study, the Tetris group played only one training game, whereas the Brain Age group played 8 types of games that included some components (e.g. calculation, reading aloud, memory). Thus, the multiple components in the Brain Age could contribute to the improvement of other cognitive functions after the training. Other possible explanations for the mechanisms of the transfer effect have been applied in previous studies. For example, previous studies have proposed the possibility of feedback processes or experiencing new things to explain the possibility the transfer effects [22]. However, these possibilities could be unavailable in our results. In our study, we recruited participants who had no experience of any video games, and employed an active control group who played the Tetris game. Thus, both groups in our study equally shared the feedback or the experience of new things from games. The difference between two training games could be effective in generating the transfer effect by the brain training game.

Can we conclude that the brain training games have a positive effect on the improvement of any other cognitions? It would be too early to accept this conclusion, because our results showed a significant transfer effect of the brain training game on only executive functions and processing speed in the elderly. In our study, we simply defined the transfer effect as improvements of untrained cognitive performance from playing the brain training games. The transfer effect also could be classified in terms of a near transfer effect and a far transfer effect [50], [51]. The near transfer effect refers to improvements in cognitive domains that are closely related to the cognitive processes trained. On the hand, the far transfer effect refers to improvements in cognitive domain that are not closely related to the cognitive processes trained. From this viewpoint, our results showed only the brain training game had the near transfer effect (improvements of executive functions and processing speed), because the training domains of the brain training game (Brain Age) would be expected to train executive functions and processing speed. There are some reasons for the absence of the far transfer effect in the present study. First, there is a possibility that the training term of our study may not be enough time to obtain the far transfer effect. Second, our measurements of cognitive functions were not appropriate to detect the far transfer effect. Most of cognitive measures in our study were classified into tasks to test the near transfer effect. In our study, there are a few tasks to detect far transfer effects. To conclude that the brain training games could improve cognitive functions, it is important to show far transfer effects from playing the brain training games. Further studies are needed to test whether or not far transfer effects as well as near transfer effects could be elicited by playing the brain training games.

There are some limitations. First limitation is that we did not measure memory performance. The prevalence of memory complaints in community-based samples of the elderly is estimated to be 25% and 50% [52]. One important future direction is to examine whether or not the brain training game would improve the memory performance in the elderly. Second limitation of our study is that we did not assess the real world task or every day cognitive abilities such as driving abilities. Future research would assess not only whether brain training game improves performance on laboratory-based tasks, but whether the brain training game improves performance on everyday cognitive abilities and real world tasks (e.g. driving skills or shopping).

## Supporting Information

Protocol S1 **Trial Protocol.**
(PDF)

Protocol S2 **Appendix 1.** Japanese version.
(PDF)

Checklist S1 **CONSORT Checklist.**
(DOC)
