# Supplementary material for: Brain Training Game Improves Executive Functions and Processing Speed in the Elderly: A Randomized Controlled Trial
Source: PLoS One. 2012 Jan 11;7(1):e29676. doi: 10.1371/journal.pone.0029676 (PMC3256163; doi:10.1371/journal.pone.0029676)
Supplement: Protocol S1 — Trial Protocol. (PDF) [file pone.0029676.s001.pdf]

The efficacy of playing the Nintendo DS games on cognitive functions of healthy elderly: a randomized controlled trial.

Trial Protocol

**Title**	The efficacy of playing the Nintendo DS games on cognitive functions of healthy elderly: a randomized controlled trial.
**Summary**	The aim of this trial is to investigate the effect of brain training games intervention (15 minutes per day, at least 5 days per week, for 4 weeks) on the cognitive functions (general cognitive function, executive function, processing speed, memory, attention).
**Study Design**	Randomized controlled trial<br>Double -blind (participants and tester blinded)
**Description**	Our cognitive functions decrease with age. These reductions of the cognitive functions make daily activity difficult for the elderly. Therefore, many researchers are interested in the method which led to improvement the cognitive functions in the elderly. Previous studies showed that cognitive trainings could improve a certain type of cognitive functions. Based on these facts, brain training games have been released. From the time brain training game, also known as Brain Age or Dr. Kawashima's Brain Training, was first released, the game was extremely popular around the world. The beneficial effects of Brain Age expected to transfer to other cognitive functions, but the beneficial effects of the brain training games are poorly understood.<br><br>**Purpose**<br>To reveal the impact of the brain training game on cognitive functions in the elderly, we plan to conduct a double-blinded randomized controlled trial.<br><br>**Participants**<br>Thirty-two participants are recruited through an advertisement in the local newspaper and screened by a questionnaire before inclusion. All participants are right-handed, native Japanese speakers, not concerned about their own memory functions, not using medications known to interfere with cognitive functions (including benzodiazepines, antidepressants or other central nervous agents), and having no diseases known to affect the central nervous system, including thyroid disease, multiple sclerosis, Parkinson's disease, stroke, severe hypertension or diabetes.<br>To maximize the benefit of the intervention, all participants are non-gamers and reported playing less than one hour of video games a week over the past 2 years.<br>To minimize the influence of subclinical degenerative conditions, the following exclusion criteria are employed. Mini Mental Status Exam (MMSE) < 26, Frontal Assessment battery (FAB) < 12 and IQ < 85 derived from the Wechsler Adult Intelligence Scale III.<br><br>**Overview of intervention**<br>The participants are asked to perform each video game training (Brain Age or Tetris) over 4 weeks with 5 training days in each week. On each training day, participants perform the video game for about 15 minutes. The participants play video

	games on the portable console, Nintendo DSi, at their homes. Game performance is recorded for each participant. At the end of each training day, participants report the scores of the played games. The Brain Age group lists the titles of trained games and a score for each trained game at the end of each training day. The Tetris group only reports the best total score at end of each training day. The measures of cognitive functions are conducted before and after training. On the first day of training (pre), all participants are tested on a series of neuropsychological and behavioral tests. After these tests, participants receive the instruction to play one of the games for 30 minutes. The following day, participants start 4 weeks video game training. After 4 weeks of training (post), all participants are re-examined on some neuropsychological and behavioral tests.<br>The procedures for this study have been approved by the Ethics Committee of the Tohoku University Graduate School of Medicine.
**Primary Outcome Measure(s)**	1)Mini-Mental State Examination (MMSE)<br>2)Frontal assessment battery at bedside (FAB)<br>3)Trail Making Test-B (TMT-B)<br>4)Digit cancellation task (D-CAT<br>5)digit span forward (DS-F)<br>6)digit span backward (DS-B)<br>7)Digit Symbol Coding (Cd)<br>8)Symbol Search (SS)
**Secondary Outcome Measure(s)**	1)Brain structure measured by MRI
**Sample Size**	Since this is the first trial of its kind in a brain training game research field, no a priori sample size estimation was carried out.
**Setting**	Sendai city, Miyagi prefecture in Japan
**Project Coordinator**	Rui Nouchi (Ph.D)<br>Researcher,<br>Institute of Development, Aging and Cancer, Tohoku University, Smart Aging International Research Center, Seiryo-machi 4-1, Aoba-ku Sendai
**Overall Study Official(s)**	Institute of Development, Aging and Cancer, Tohoku University, Smart Aging International Research Center, Seiryo-machi 4-1, Aoba-ku Sendai
**Human Subjects Review/Oversight**	This research was approved by the Ethics Committee of the Tohoku University Graduate School of Medicine.
**Collaborators**	Ryuta Kawashima[1,2,3], Yasuyuki Taki[2], Hikaru Takeuchi[1], Hiroshi Hashizume[2], Yuko Akitsuki[1], Yayoi Shigemune[3], Atsushi Sekiguchi[3], Yuka Kotozaki[1], Takashi Tsukiura[3], Yukihito Yomogida[3,4].

	1 Smart Ageing International Research Centre, Institute of Development, Aging and Cancer, Tohoku University, Sendai 980-8575, Japan<br>2 Division of Developmental Cognitive Neuroscience, Institute of Development, Aging and Cancer, Tohoku University, Sendai 980-8575, Japan<br>3 Department of Functional Brain Imaging, Institute of Development, Aging and Cancer, Tohoku University, Sendai 980-8575, Japan<br>4 Japanese Society for the Promotion of Science, Tokyo102-8472, Japan
**Funding Source**	The Research Institute of Science and Technology for Society/ Japan Science and Technology Agency
