# Supplementary material for: Brain Training Game Improves Executive Functions and Processing Speed in the Elderly: A Randomized Controlled Trial
Source: PLoS One. 2012 Jan 11;7(1):e29676. doi: 10.1371/journal.pone.0029676 (PMC3256163; doi:10.1371/journal.pone.0029676)
Supplement: Protocol S2 — Appendix 1. Japanese version. (PDF) [file pone.0029676.s002.pdf]

（平成２１年４月１日施行様式）

実　施　計　画　書

Ⅰ　課題名　各種エンタテインメントが認知機能に与える影響の研究

Ⅱ　研究等の概要（必要に応じ倫理審査の判断材料になる論文・資料・調査票などを添付する。専門外の委員、外部委員が研究内容を短時間に把握できるように記述を工夫する。）

＜研究の意義･目的＞（研究の背景や倫理的側面を含める。）

エンタテインメントは、人工的且つ合法的で全年齢または特定の年齢層の人々の感情に働きかけ、何らかの感動を起こさせ「楽しむこと」を目的にした行為や催し物などのことであると定義される。エンタテインメントは、現代社会において、心豊かな生活のために必要欠くべからざるものである。

代表的な室内型エンタテインメントとしては、テレビはほぼ全ての家庭にあり毎日のように視聴されており、またテレビゲームも日本人の４人に一人が家庭で日常的に行っているとされている。これらのエンタテインメントは、人間の脳と心に直接働きかけるものであり、ライフスタイルに影響を与えることもある、きわめて強力なインタフェースであるが、それらが人間に与える影響は科学的にはほとんど解明されていない。

本研究の目的は、ゲーム、テレビ、映画などのエンタテインメントが心身に与える影響を科学的に解析・評価をおこなうことである。特に、人が加齢による経年変化に賢く対処し、個人・社会が知的に進化するために、エンタテインメントとどう関わるべきか、もしくは、そのようなスマートエイジング社会を構築するためにエンタテインメントはどうあるべきかを脳機能イメージングと認知心理学的手法によって検証する。

本研究では、心身機能測定、MRI 測定を行うが、ともに非侵襲的手技である。MRI 撮影に関しては、後述の如く米国ＦＤＡ基準を満たしたシークエンスのみを用いることから、研究手技上倫理的に問題を生じる可能性はないと考えている。また、エンタテインメント介入（テレビ、映画、音楽、ゲームなど）は、視覚刺激を用いており、光過敏性発作を起こす可能性については、後述のごとく国際電気通信連合の指針を準拠することで対応ため、倫理的問題を生じる可能性は殆どないと考えている。

＜対　象＞（研究に必要な概略の対象人数等の数値を記すこと。）

脳血管障害の既往、脳外傷の既往、てんかんやけいれんの既往、5分以上の意識消失の既往、悪性腫瘍の既往、精神疾患の既往等がなく健康に日常生活を送っている右利き成人を対象とする。５年間の研究期間内に２０～３０歳代の若年層被験者約１００名、４０～５０歳代の中年層被験者約１００名、６０～７０歳代の壮年層被験者約１００名を対象とする。

＜実施計画＞(多施設共同研究の場合には、全体計画と本学の役割分担について分かるように記入する。また記入上の注意に従い必要書類を添付する。)

ヘルシンキ宣言に則り被験者に研究の目的、安全性について書面、及び口頭にて説明したのち、書面による同意書を得る。

今回の研究で用いるエンタテインメントは、テレビ、映画（ＤＶＤ）、音楽、テレビゲームの４種類とする。テレビ、映画（ＤＶＤ）、音楽は、被験者に予めそれらのエンタテインメントに関する趣向をアンケート調査しておき、好みのジャンルのものと、好みでも嫌いでもない中立なジャンルのものの２種類を準備する。テレビゲームは、全ての年齢層の被験者が実施可能なできるだけ操作の易しいゲームを、シューティングゲーム、パズルゲーム、学習ゲーム、バイオフィードバックゲームの４つのカテゴリーから準備する。

表１　研究に用いるエンタテインメントの種類

分類	下分類			
テレビ	好みのもの	中立のもの		
映画（ＤＶＤ）	好みのもの	中立のもの		
音楽	好みのもの	中立のもの		
テレビゲーム	シューティング	パズル	学習	バイオフィードバック

各種エンタテインメントが認知機能と脳活動に与える短中期的影響の検討

被験者は、エンタテインメントやテレビゲームを、それぞれ１日１５分、週に５日間、４週間にわたり自宅で継続して行う。その際の脳機能、脳形態、認知機能の変化、気分の変化を、表２のスケジュールにそって計測する。研究中の被験者の日常生活上のエンタテインメント経験に関しては、事前にアンケート調査を行い、被験者間で隔たりがでないよう統制する。

表２　研究２のスケジュール

スケジュール	内容
事前説明	認知機能検査
１日目	脳ＭＲＩ検査、認知機能検査、精神ストレス度検査
２日目～２８日目	Ａ群もしくはテレビゲーム
２９日	脳ＭＲＩ検査、認知機能検査、精神ストレス度検査

脳ＭＲＩ検査は、加齢医学研究所の 3 テスラ MR スキャナー（フィリップス社製　アチーバ 3.0T Quasar Dual）を用いて行う。全ての被験者の、脳形態ＭＲＩ撮像、脳機能ＭＲＩ撮像、脳灌流 MRI、拡散 MRI 撮像を、すでに東北大学医学部・医学系研究科倫理委員会承認を得て研究を開始している「MRI を用いた成人の脳形態、脳機能、認知機能に関する研究」と、全く同一のプロトコールで行う。

認知機能検査は、大脳の前頭葉、頭頂葉、側頭葉の各連合野機能を調べるテスト形式調査用紙によって行う。この認知機能検査は、すでに東北大学医学部・医学系研究科倫理委員会承認を得て研究を開始している「学習障害の脳機能コホート研究」で用いているものと全く同じものを用いる。

＜使用する研究費＞（必ずどれかを選択。）
■運営交付金　　■科学研究費　　□厚生労働科学研究費
□その他公的研究費（　　　　　　　　　　　）
□寄附金・研究助成金　□共同研究費（　　　　）、受託研究費（　　　　　）
□その他（知財創造経費）

Ⅲ　研究等の実施場所

東北大学加齢医学研究所ブレインイメージング研究棟

Ⅳ　実施に際しての倫理的配慮について

Ⅳ－１＜研究等の対象とする個人の人権への対策＞

（プライバシー確保の方途その他について具体的に記すこと。）

本研究は人間を対象とした医学研究のガイドライン（ヘルシンキ宣言）に準拠して行う。被験者（加えて未成年者にはその保護者）には研究の目的、方法、予想される危険性に関して十分な説明を行う。具体的には MRI 撮像にて予想されうる危険性、更には被験者から得られたデータは、教育、研究にて公表されるが、個人を特定できる情報は一切公表しないこと、この研究への参加に同意した場合でも随時これを撤回でき、そのことによる不利益は何等受けることはないことを詳細に説明する。問診、及びMRI撮像に先立って、書面によるinformed consent を被験者から得る。

個人を特定可能な情報（連結表、アンケート調査、同意書、謝金書類など）は、施錠可能な保管庫に厳重に保管する。脳画像はインタネットに接続をしない環境下でメディアに保管し、パスワードで管理する。画像は、ヘッダーから個人情報部分を消去、顔を同定可能な軟部組織状況を専用ソフトにより消去したものを研究用に用いる。

また、被験者はいかなるときでも研究を中断できる事、訓練セッションにおいては自分のペースで適宜休憩を入れる事ができる事をあらかじめ伝えておく

Ⅳ－２＜対象者を選ぶ方針･基準＞

（健常者を対象にする場合は公募の形式にすること。）
謝金等　　□無　　☑有

20 歳～79 歳の健康な右利き成人を公募する。拘束時間に相当する研究者協力謝金を支払う。

Ⅳ－３＜個人情報の取り扱いについて＞

（当該臨床研究に係る個人情報の保護の方法等又は委託先における個人情報の監督体制等について具体的に記すこと。また、多施設共同研究の場合、他機関と本学の関係、データセンター等についても言及すること。）

1）個人情報管理施設が東北大学大学院医学系研究科に……□該当する
☑該当しない

2）個人情報管理施設が東北大学大学院医学系研究科に該当する場合

管理者氏名：

資格（医師、臨床検査技師等）：

（委嘱される場合、守秘義務が生じる）

3) 匿名化の有無..................................☑匿名化する　　　□匿名化しない

4) 匿名化する場合.......................☑連結可能匿名化　□連結不可能匿名化

5) 連結可能匿名化する場合は連結表の管理方法を具体的に記載：施錠可能な保管庫に厳重に保管する。管理者氏名：川島隆太（医師）

6) 匿名化しない場合は個人情報保護の方法を具体的に記載：＿＿＿＿＿＿＿＿

7) 個人情報管理施設が東北大学以外にある場合の個人情報管理者氏名・所属と個人情報保護を具体的に記載：＿＿＿＿＿＿＿＿＿＿＿＿＿＿＿＿＿＿＿＿

Ⅳ－４＜対象者に理解を求め同意を得る方法＞（下記の該当項目を選択する）

対象者各人に（**□1.書面のみ　□2. 口頭のみ　☑3. 書面と口頭**）で説明した後

☑A.　対象者の署名入りの同意書を保管する。

□B.　対象者の同意の署名が記された診療録を保管する。

□C.　対象者の同意の署名が記された調査票を保管する。

※対象者に対する説明同意文書と同意文書を必ず添付する。健常者、患者、専門外の委員、外部委員が実施内容を短時間に把握できるように記述を工夫する。

Ⅳ－５＜対象者が未成年者の場合、成年者でも十分な判断力のない場合又は病名に対する配慮が必要な場合などにおける対処方法。＞

（下記の該当項目を選択する。複数選択可。B-F の場合は対処方法及び対象とする理由を具体的に記載する。）

☑A　下記特例を対象にしない（対処方法の記載の必要がない）

□B　未成年者

□C　十分な判断力がない成年者

□D　意識のない成年者

□E　病名に対する配慮が必要な成年者

□F　その他

B-F の者を研究対象とする理由

具体的な対処方法：（下記の該当項目を選択。複数選択可。例外の場合は対処方法を具体的に記載。）

□インフォームド・アセントを取得する。

□保護者・代諾者の署名入りの同意書を保管する。

□保護者・代諾者の同意の署名が記された診療録を保管する。

□保護者・代諾者の同意の署名が記された調査票を保管する。

Ⅴ　期待される研究成果あるいは予測される利益

本研究の直接の成果によって、人が加齢による経年変化に賢く対処し、個人・社会が知的に進化するために、エンタテインメントとどう関わるべきか、もしくは、そのようなスマートエイジング社会を構築するためにエンタテインメントはどうあるべきかについての、科学的エビデンスを持った知識を得ることができる。

この研究成果を社会に情報発信することによって、スマートエイジング社会を創生し、持続可能な循環型社会の構築に寄与する。

Ⅵ　予測される危険と不利益とそれに対する配慮・補償

Ⅵ－１＜研究等によって対象者に生じうる危険と不快に対する具体的配慮＞

（箇条書きで記し、それぞれに対する配慮の内容も記すこと。）

１．ＭＲＩ検査：

医師による問診により、頭部手術歴、体内金属の有無、ペースメーカ装着の有無、閉所恐怖などがないことを、事前に確認し、検査直前にも金属を身に付けていないことを確認する。検査中に不快を感じた場合には、いつでも検査を中断できることを予め伝えておく。３ＴＭＲＩ装置を用いた脳機能マッピングの安全性に関しては米国ＦＤＡ基準を満たしたシークエンスのみを用いる。

２．エンタテインメント：

テレビ、ビデオの視聴、テレビゲームを行うことにより、それらはディスプレイ注視を持続的に行わせる課題である事から、被験者の体質・当日の体調によっては光過敏性発作を起こす可能性が考えられる。光過敏性発作の誘発に関しては一般的に十ー数十 Hz の繰り返し映像が最も危険とされており、国際電気通信連合の指針に基づき３Hz 以上の繰り返し映像を避けるとする規制がなされており、それに準拠する。また、被験者が練習時・研究時ともに体調の不良・不快を感じた場合、直ちに研究を中止するよう配慮を行う。

Ⅵ－２＜対象者に健康被害が生じた場合の補償の有無及び具体的な措置＞

研究の種類を選択すること

□①介入を伴う研究であって、医薬品又は医療機器を用いた予防、診断又は治療方法に関するもの（対外診断を目的とした研究を除く）

「補償のための保険その他の必要な措置」について、事前に十分な説明を行い、対象者の同意を受けなければならない。

☑②それ以外の介入を伴う研究（体外診断を目的とした研究を含む）

補償の有無を説明する必要がある。

□③観察研究にあたっては、試料等の採取が侵襲性を有する場合

補償のための保険等必要な措置の有無等を対象者に十分に説明する必要がある。

補償の種類を選択すること

☑補償のための保険を設定した

□補償のための保険を設定する予定である（具体的内容：　　　　　　　　　　　　　　　　　）
□保険以外の対処方法を講じた（具体的内容：　　　　　　　　　　　　　　　　　　　　　）
□補償がない旨を説明する

措置内容
☑説明同意文書に補償のための保険等必要な措置について記載した
□補償のための保険等必要な措置を証明する文書を添付した

Ⅶ　研究期間終了後の試料の取扱い

□　廃棄する
☑　保存する
（保存するを選択した場合は下記について記載すること。）
ア　試料等の名称：脳ＭＲＩ画像
イ　試料等の保管場所：加齢医学研究所脳機能開発研究分野
ウ　試料等の管理責任者：川島隆太
エ　被験者等から得た同意の内容（将来にわたって試料を解析する場合はその旨を対象者に説明し同意を得ること）：研究期間終了後も脳画像データに関しては個人情報を特定されない形で再解析用として保存し研究目的にて使用する。

匿名化の方法
□連結可能匿名化（理由：　　　　　　　　　　　　　　）

☑連結不可能匿名化

Ⅷ　備　考

（企業等法人との利益相反、特許申請等が絡んでいる場合あるいは，その他の利害関係がある場合についてもその旨記入すること。また、介入を伴う研究であって侵襲性を有するものについて、公開されているデータベースに臨床研究登録しない場合には、登録により臨床研究に著しい支障が生じる理由を明記すること。）

利益相反（当該企業等との利害関係）について
　主任研究者等は、本研究の実施に際し、利益相反マネジメント委員会の審査と承認を得ている。また、主任研究者等は、本研究の経過を定期的に東北大学利益相反マネジメント委員会へ報告等行うことにより、本研究の企業等との利害関係についての公正性を保つ。

（注１）　紙面がたりない場合は、別紙に記入すること。

説明書

研究課題名：エンタテインメントが認知機能に与える影響の研究

○はじめに

この説明は、予めMRI検査のことを正しく理解した上で、あなた自身の自由な意志に基づいてこの研究に協力して頂けるかどうか判断して頂くためのものであり、人間を対象とした医学研究のガイドライン（ヘルシンキ宣言）に準拠しています。

以下にこの研究の目的や調査の内容について説明します。不明な点があればどんなことでも遠慮なく質問して下さい。

○研究の目的

本研究の目的は、ゲーム、テレビ、映画などのエンタテインメントが心身に与える影響を科学的に解析・評価をおこなうことにあります。特に、人が加齢による経年変化に賢く対処し、個人・社会が知的に進化するために、エンタテインメントとどう関わるべきかを脳機能イメージングと認知心理学的手法によって検査・検証します。

○研究代表者（責任者）

東北大学加齢医学研究所　脳機能開発研究分野　教授　川島隆太
連絡先　980-8575 仙台市青葉区星陵町4－1
東北大学加齢医学研究所　脳機能開発研究分野
電話・ファックス　022－717－7988

○検査の方法

◆認知機能調査

まずご家庭で、生活習慣、現病歴、ご自身の性格、趣味などに関するアンケート用紙を記載していただきます。

次に東北大学にて来ていただき、面接にて心の働きを調査します。この面接調査には約 1 時間半程度かかります。一部のアンケート調査と面接調査は、後述する2回目のMRI検査の時にも行います（計3回）。

◆MRI検査

指定の日時に東北大学加齢医学研究所ブレインイメージング研究棟に集合していただきます。MRI検査は、この日、その4週間後（2回目）の計 2 回行います。MRI撮像には費用はかかりません。

MRI 撮像は、磁場が発生している装置に入って、静かに動かずに横になって頂いて行います。MRI 撮像時間は、出入りも含め約 1 時間程度です。最初の 20 分程度は何もせずに横になっていただき、脳の形態、血流量（1 分当たり脳にどの程度の血液が流れているか）を MRI にて測定します。今回行う MRI 検査は、脳の血管障害、脳腫瘍などの脳の病気を見つけるのに非常に適した方法です。この MRI 検査では現在のところ重大な副作用の報告はありません。しかし、強力な磁場の発生している部屋に入っていただくため、心臓にペースメーカーを装着されている方、脳動脈クリップを用いた治療を受けた方、体内に金属を入れる手術を行った方は MRI を受けることが出来ませんので申し出て下さい。また、妊娠初期の胎児への影響は完全には解明されていないため、妊娠初期の方も受けることが出来ません。閉所恐怖症の方も MRI を行うことは出来ません。

本研究で撮像する MRI は診断のためのものではありません。また、今回の撮像条件は微小な脳腫瘍や脳梗塞などを発見するための撮像法は含まれておらず小病変の検出は困難です。さらに、脳血管の評価を行える条件では撮像しないため、脳動脈瘤などの脳動脈に関する評価は行えません。本研究で撮像する MRI はあくまで研究目的のためのものであり，脳検診のような診断を目的としたものではないことを，ご承知おき下さい。

◆エンタテインメントの家庭での実施

第1回目と第2回目のMRI検査の間の4週間にわたり、ご家庭で、指定されたエンタテインメント

(ビデオ、音楽、テレビゲーム)を、1日15分間、週に5日間楽しんでいただきます。エンタテインメントの行い方などについては、MRI検査時に詳細に説明をいたします。毎日のエンタテインメントを行っていただいた時間を指定のメモ用紙に記録していただきます。

体調のすぐれない時や、お仕事などで忙しい時には、無理をなさらなくて結構ですし、また行う時間もご自身の都合のよい時間帯で結構ですが、できるだけ1週間に5日間行えるよう意識をしてください。

○あなたにもたらされる利益と不利益

◆利益

研究にご協力いただく御礼として、実験により拘束された時間に相当する研究者協力謝金(大学規定額)をお支払いたします。

◆不利益

MRI検査では、磁力を使って人体の内部を画像化する技術であり、放射線被爆はありません。また、一般の臨床検査にも用いられており十分安全性が確立されています。しかし、以下に該当する方は研究の安全性の問題から研究に参加いただけません。

①体内に金属が存在する方(脳動脈瘤クリップ、ペースメーカー、骨折治療後のボルトやプレート、歯の矯正治療中、髪染後1ヶ月以内、入れ墨など)

②極度の近視、聴力障害、閉所恐怖症、暗所恐怖症の方

テレビ、ビデオの視聴、テレビゲームを行うことにより、視覚刺激の点滅などによって、ごくまれに気分がすぐれなくなったり、疲労やめまいを感じたり、光過敏性てんかんを発症したりすることがあります。この場合には、テレビ、ビデオの視聴、テレビゲームをただちに中止していただき、研究代表者まで電話等でお申し出ください。医師の診察が必要と判断された時には、医療機関を紹介いたします。

○健康被害の補償について

本研究において、有害事象が発生した場合、MRI撮像に関わる事故に関しては障害保険が適応されます。

○個人情報の保護

あなたの個人情報の秘密は厳重に守られます。得られた画像等のデータは研究や教育の目的に使用されたり、論文等の形で公表されることがあります。しかし、氏名など個人を特定できる情報は一切公表されません。

○利益相反(企業等との利害関係)について

本研究は東北大学の主任研究者のグループにより公正に行います。本研究の企業との利害関係については、東北大学利益相反マネジメント委員会の審査と承認を得ております。また、本研究の経過を定期的に東北大学利益相反マネジメント委員会へ報告等行うことにより、本研究の企業等との利害関係についての公正性を保ちます。

○研究成績の公表

ご協力により得られた成績は、個人が特定できないようにした上で統計処理を行い、エンタテインメント、もしくは医科学関連の学会での発表および医科学雑誌への投稿の形で公表することを予定しています。

○研究期間終了後の検査データの取扱い

研究期間終了後も脳画像データに関しては個人情報を特定されない形で保存し、再解析用として研究目的にて使用することがあります。

○同意書、アンケート調査用紙の保管

この研究でご提出いただいた同意書およびアンケート調査用紙は、東北大学加齢医学研究所脳機能開発研究分野に研究終了まで厳重に施錠保管いたします。管理責任者は加齢医学研究

所脳機能開発研究分野　教授　川島隆太です。なお承諾の取り消しがあり次第、承諾書は消却処分いたします。

○任意の同意と同意の撤回

この研究に参加するかどうかは、あなたの自由な意思で決めて下さい。ご協力が得られなくてもあなたの不利益になることは全くありません。また、ここで同意していただいても、その同意を後日取り消すこともできます。ただし同意を取り消す前にすでに報告された研究結果について公表を取り消すことはできませんが、同意を取り消した後はあなたの研究結果は廃棄され使用されることはありません。

同意を取り消す場合には協力者ご本人か本人から請託を受けた代理人が次のページに用意しました同意を取り消す旨の書面を下記宛にお送りくださるよう御願いします。

送付先　〒980-8575　仙台市青葉区星陵町４－１
東北大学加齢医学研究所脳機能開発研究分野
川島隆太　宛

最後に、何卒皆様の御理解と御協力を賜りますよう、よろしくお願い申し上げます。

東北大学大加齢医学研究所脳機能開発研究分野
川島隆太

尚、御不明な点、ご質問、お問い合わせ等ございましたら、下記までご連絡下さい。

〒980-8575　仙台市青葉区星陵町４－１
加齢医学研究所プロジェクト棟1階
東北大学加齢医学研究所脳機能開発研究分野
川島隆太
電話・ファックス　022－717－7988
電子メール　fbi@idac.tohoku.ac.jp

「エンタテインメントが認知機能に与える影響の研究」参加の同意書

東北大学　加齢医学研究所
所長　福田　寛　殿

上記について別紙（「エンタテインメントが認知機能に与える影響の研究」に関する研究に被験者として参加するにあたっての同意文書）を読み、口頭でも説明を受け、検査の目的、方法、安全性について十分理解できましたので、この研究に被験者として参加することに同意致します。

フリガナ
平成　　年　　月　　日　　　　　　被験者署名　　　　　　　　　（男・女）　印

（被験者が未成年者の場合）　　　　　　　フリガナ
平成　　年　　月　　日　　　　被験者保護者署名　　　　　　　（男・女）　印

住　　所

電話番号　　　　　　　　　　　　　　生年月日　　　年　　　月　　　日

説明者：東北大学加齢医学研究所　脳機能研究開発分野
実施責任者：東北大学加齢医学研究所　川島隆太
連絡先：〒980-8575 仙台市青葉区星陵町 4-1
東北大学加齢医学研究所　脳機能開発分野
TEL/FAX 022-717-7988

「エンタテインメントが認知機能に与える影響の研究」に

関するMRI 実験にあたっての同意書

東北大学　加齢医学研究所
所長　福田　寛　殿

上記について別紙（「エンタテインメントが認知機能に与える影響の研究」に関する研究のためのMRI 実験に参加される方への説明同意文書）を読み、口頭でも説明を受け、検査の目的、方法、安全性について十分理解できましたので、この検査を受けることに同意致します。

また、実験中の万が一の事故に対して傷害保険を適用させるために、当研究室が契約を結んでいる保険会社の傷害保険に加入します。その際、自分の氏名、住所、生年月日が、上記保険会社に提出されることに同意します。

また、実験用の脳画像で異常が疑われる所見が発見された場合、その事実について、医師からの告知に

同意します　・　同意しません
（どちらかを○で囲んでください）

フリガナ
平成　年　月　日　　被験者署名　　　　（男・女）　印

（被験者が未成年者の場合）　　フリガナ
平成　年　月　日　　被験者保護者署名　　　　（男・女）　印

住　所

電話番号　　　　生年月日　　年　　月　　日

説明者：東北大学加齢医学研究所　脳機能研究開発分野
実施責任者：東北大学加齢医学研究所　川島隆太
連絡先：〒980-8575 仙台市青葉区星陵町 4-1
東北大学加齢医学研究所　脳機能開発分野
TEL/FAX 022-717-7988

MRI 実験参加直前チェックシート

(I)　下記の質問事項をよく読んで、「はい」、「いいえ」もしくは「わからない」のどれかに丸をつけてください。「はい」もしくは「わからない」があった場合は、実験責任者が詳細を確認させていただき、実験に参加できるかを判断します。

1. 現在通院中ですか。　はい　いいえ
2. てんかん発作を起こしたことがありますか。　はい　わからない　いいえ
3. 体温の調節または発汗能力に障害がありますか。　はい　わからない　いいえ
4. 不整脈はありますか。　はい　いいえ
5. 発声、聴覚または握力に障害がありますか。　はい　いいえ
6. 計測実験中のコミュニケーションに問題があると予想されますか。　はい　いいえ
7. 入れ歯、義歯、義眼、義足、ギプスなどを使用していますか。　はい　いいえ
8. 歯科矯正用ブリッジをつけていますか。　はい　いいえ
9. アイラインやマスカラなど金属性顔料を含む化粧品を使用していますか。　はい　わからない　いいえ
10. 1か月以内に髪を染めましたか。　はい　わからない　いいえ

(II)　下記の質問事項をよく読んで、「はい」、「いいえ」もしくは「わからない」のどれかに丸をつけてください。これらのうち一つでも「はい」または「わからない」があった場合は、MRI 室には入ることができないため実験には参加できません。

1. 心臓ペースメーカー、または心臓の人工弁をお持ちですか。　はい　わからない　いいえ
2. 体内、または体外の生命維持装置に依存していますか。　はい　わからない　いいえ
3. 頭蓋内に金属性の動脈瘤クリップをお持ちですか。　はい　わからない　いいえ
4. 止血のための外科用クリップや強磁性体を体内にお持ちですか。　はい　わからない　いいえ
5. 金属性のピン、棒等による骨の治療を受けていますか。　はい　わからない　いいえ
6. 人工内耳の埋め込みを受けましたか。　はい　わからない　いいえ
7. リザーバー(皮下埋め込み型薬剤注入器具)、カテーテル等の各種金属製チューブ、
ステープル（医療用の縫合用ホッチキス）、関節/四肢プロテーゼ（医療用の体内埋入物）、
インスリンポンプ、神経刺激装置、脳脊髄液短絡術用圧可変式シャント、
人工関節、体内避妊具などの体内金属をお持ちですか。　はい　わからない　いいえ
8. 体内に金属片のある可能性がありますか
（兵役時に受けた銃弾、その破片、眼球内異物混入の可能性含）　はい　わからない　いいえ
9. 閉所恐怖反応を起こしたことがありますか。　はい　いいえ
10. 入れ墨（まゆ入れ墨を含む）をしていますか。　はい　いいえ
11. 現在、妊娠している可能性がありますか。　はい　わからない　いいえ

(III)　下記の質問事項をよく読んで、「はい」、「いいえ」もしくは「わからない」のどれかに丸をつけてください。これらは当日の注意事項です。下記の状態で MRI 室に入らないように気をつけてください。

1. コンタクトレンズをつけていますか。　はい　いいえ
2. 湿布などを身体に貼り付けている、
もしくは経皮吸収型・虚血性心疾患治療剤を使用していますか。　はい　わからない　いいえ
3. ポケットの中に、財布・携帯電話・鍵・カードなどが入っていますか。　はい　いいえ
4. 金属類を身に着けていますか（ネックレス、ピアス、ヘアピン、腕時計、
ワイヤーの入った下着、ピップエレキバン、カイロ、衣類付属の金属など）。はい　いいえ

以上の質問内容をよく理解した上で答えました。

記入日(実験日):　　　年　月　日

生年月日:　　　年　月　日　性別:(男・女)

住所:

被験者署名:

実験実施者への注意:

上記(II)でひとつでも、「はい」もしくは「わからない」に記入した被験者は、実験参加不可。(III)で、ひとつでも、「はい」もしくは「わからない」に記入した被験者は、当該物品を外せれば実験可。(IV)で、「同意しません」に記入の被験者も参加不可。上記(I)で、「はい」もしくは「わからない」に記入した被験者は、実験の責任者(安全責任者)の確認ができた場合を除いて、実験参加不可。確認できた場合は確認者の氏名を以下に記入すること。

確認者(安全責任者)氏名

「エンタテインメントが認知機能に与える影響の研究」参加の同意の撤回書

私　　　　　　　　　　　　は、東北大学大学院加齢医学研究所脳機能開発分野が実施した「エンタテインメントが認知機能に与える影響の研究」（代表者　川島隆太）に協力することを同意しましたが、この同意を撤回します。

平成　　年　　月　　日

同意者氏名＿＿＿＿＿＿＿＿＿＿印　（ご本人のご署名または記名捺印）

同意者住所＿＿＿＿＿＿＿＿＿＿＿＿＿＿＿＿

（代理人の場合）
代理人氏名＿＿＿＿＿＿＿＿＿＿＿＿＿＿＿印

代理人住所＿＿＿＿＿＿＿＿＿＿＿＿＿＿＿＿
